# Deficit irrigation in a young, super high-density hedgerow olive orchard (cv. Arbosana): effects on physiological, vegetative, and productive parameters

**DOI:** 10.3389/fpls.2026.1814138

**Published:** 2026-06-11

**Authors:** Luciana Gentili, Martin Tivani, Valerio Mastio, Flavio Capraro, Cibeles Contreras, Mariela Torres, Damián Maestri, Pierluigi Pierantozzi

**Affiliations:** 1 Estación Experimental Agropecuaria San Juan, Instituto Nacional de Tecnología Agropecuaria (INTA), Consejo Nacional de Investigaciones Científicas y Técnicas (CONICET), San Juan, Argentina; 2 Instituto de Automática, Universidad Nacional de San Juan, Consejo Nacional de Investigaciones Científicas y Técnicas (CONICET), San Juan, Argentina; 3 Instituto Multidisciplinario de Biología Vegetal, Consejo Nacional de Investigaciones Científicas y Técnicas, Universidad Nacional de Córdoba, Córdoba, Argentina

**Keywords:** olive, cv. ‘Arbosana’, super high-density growing system, regulated deficit irrigation, sustained deficit irrigation, water productivity

## Abstract

Over the past three decades, super high-density (SHD) olive orchards (>1000 trees ha⁻¹) have been widely implemented for olive (*Olea europaea* L.) cultivation. These systems enable early entry into production and full mechanization, but require high-water supply and they do not tolerate prolonged water stress. These latter aspects represent a challenge for arid growing areas such as those in western Argentina. This study evaluated several productive, vegetative and physiological responses to deficit irrigation (DI) strategies in a young SHD orchard (cv. Arbosana). Measurements were made during the first three productive seasons. Three DI treatments - one sustaining DI at 70% ETc throughout the entire growth period evaluated (CDI70), and two regulated DI treatments applying 50% and 25% of ETc (RDI50 and RDI25, respectively) during non-critical periods - were compared against a control (100% ETc) treatment. The total irrigation water savings were 30% (CDI70 and RDI50) and nearly 50% (RDI25). No clear differences in vegetative parameters were found between the different DI treatments, and between these and the control treatment. In contrast, fruit yield parameters showed a marked effect of the different DI treatments on alternate bearing of the olive crop. This was particularly noticeable for the two regulated DI treatments, in which a significant decline in yield was observed in the second crop cycle. Differently, the continuous DI treatment (CDI70), besides maintaining a balanced water status, was able to sustain and significantly increase fruit and oil yield at the end of the third crop cycle. In this latter treatment - considering the cumulative results of the three cycles examined - those yield parameters also showed significantly higher values than those from the control treatment. As a result of lower water consumption and higher yields, water productivity was also significantly enhanced. Because of major effects on fruit bearing, moderate or severe water restrictions (RDI50 and RDI25 treatments) would not be viable for sustaining production of 'Arbosana' trees in SHD systems. Although the results must be validated in longer trials, continuous DI (70% ETc during the entire crop season) could moderate vegetative growth and increase productivity in a sustained manner.

## 1 Introduction

Argentina stands as the leading olive producer outside the Mediterranean region (Torres et al., 2017). Over the past decade, interest in olive cultivation has resurged, largely driven by the sharp increase in international oil prices. New plantations have embraced the principles of modern olive growing, characterized by high planting density, systematic fertilization, and the widespread adoption of drip irrigation. In line with global trends, a significant proportion of these plantations have been established as high-density hedgerows, designed to ensure early production and enable full mechanization of harvesting and, in some cases, pruning operations (Famiani et al., 2023).

The super-high-density (SHD) olive cultivation model - characterized by planting densities exceeding 1000 trees per hectare - was specifically developed to accommodate over-the-row harvesters. This mechanization significantly reduces harvesting costs when compared not only to manual methods but also to the use of trunk shakers (Farinelli and Tombesi, 2015).

Efficient harvesting with over-the-row machinery requires olive trees to be planted in rows that form continuous foliage walls (hedgerows), making them compatible with mechanized systems. Each tree must be fully accommodated within the harvester head, which necessitates adapting the grove’s structure, morphology, and physiological traits to the machine’s operational requirements (Connor et al., 2014).

The varieties planted in these systems should be early-bearing and exhibit low vigor. A limited number of cultivars meet these characteristics (Rosati et al., 2024). On the other hand, SHD systems require substantial irrigation from the moment of establishment, due to both the extensive vegetative cover - which increases water consumption - and the need for rapid canopy development to secure early yields. Once maturity and the desired canopy volume are achieved, high-density hedgerow olive groves are thought to consume no more water than fully mature intensive groves (Orgaz et al., 2006). Nevertheless, root exploration capacity differs markedly between systems: in high-density hedgerows, the soil volume accessed by roots is considerably smaller, reducing the grove’s resilience to prolonged drought. Consequently, the long-term success of high-density hedgerow plantations is highly dependent on reliable water availability (Sobreiro et al., 2023).

The availability of water for irrigation is a growing concern for olive production, particularly in arid and semi-arid climates such as those prevailing in olive growing regions in north-western and central-western Argentina (Battistella et al., 2023; Villarroel et al., 2026; Wang et al., 2025). In these regions, variations in both quantity and distribution of rainfall, together with high temperatures and the resulting increase in crop evapotranspiration, generally demand greater irrigation inputs than those required in many Mediterranean growing regions (Torres et al., 2017). Elevated temperatures stimulate vegetative growth, while limited rainfall demands continuous irrigation. If this is combined with poor irrigation scheduling, it can result in excessive canopy development, which in turn makes mechanized harvesting difficult, and increases pruning requirements, ultimately diminishing yields in the current season (Albaracín et al., 2017).

Regulated deficit irrigation (RDI) represents an appropriate strategy for optimizing water use in olive cultivation. This approach adjusts irrigation inputs to the physiological state of the tree, reducing water supply during phenological stages where controlled deficits do not significantly affect yield or quality, while fully meeting crop demand during the rest of the cycle. Successful implementation of RDI requires detailed knowledge of the tree’s responses to sub-optimal water availability at each developmental stage. Previous studies have highlighted the olive tree’s heightened sensitivity to water stress during pre-flowering and flowering (Rapoport et al., 2012; Pierantozzi et al., 2013, 2014), the initial phase of pit hardening, and the onset of fruit ripening (Fernández et al., 2013). Fernández et al. (2020) reported that RDI can reduce irrigation requirements by up to 60% without significantly compromising yield or oil quality. Similarly, in super-high-density (SHD) olive groves, controlled water stress has been shown to exert minimal or even beneficial effects on oil yield, while enhancing oil quality (Trentacoste et al., 2019). Despite these advances, few studies have explored strategies that integrate sustained and regulated deficit irrigation across multiple phenological phases within a single trial throughout the entire crop cycle. In arid olive growing regions with very low rainfall - such as those at San Juan province in Argentina - where olive cultivation is feasible only under irrigation, unique opportunities exist to evaluate and compare irrigation strategies without interference from precipitation. This study evaluated several productive, vegetative and physiological responses to deficit irrigation (DI) strategies in a young SHD orchard (cv. Arbosana). We anticipate observing substantial differences in vegetative growth and productivity in olive trees under DI compared to trees without water deficit. We also hypothesize that a sustained DI regime may decrease vegetative growth and maintain or even increase productivity parameters compared to a fully irrigated treatment.

## 2 Materials and methods

### 2.1 Field trial and plant material

The field trial was conducted in an olive orchard located at the Agricultural Experiment Station (EEA - INTA San Juan), San Juan province, Argentina (31°39′32.57″ S; 68°35′22.82″ W). The soil type of the study site corresponds to Entisol of the El Salado Complex, being 0.85 m in depth and having 36 mm of water-holding capacity (Babelis et al., 2013). Soil texture is loam in the upper 40 cm and sandy loam between 0.4 and 0.8 m. The soil is slightly alkaline (pH 7.9) and poor in organic matter (0.9% at 0–20 cm depth; 0.5% at 20–80 cm). Nutrient contents range between 600–800 ppm nitrogen, 50–80 ppm phosphorus, and 150–300 ppm potassium.

Fertilization consisted of three annual foliar applications between early spring and summer, complemented by fertigation based on annual foliar analyses conducted each December. Pest and disease management followed standard local practices, using preventive foliar applications with products of different modes of action. Mechanical side pruning was simulate in winter with a hedge trimmer, alternating the pruned side each year. At the start of each growing season, row width ranged between 1.3 and 1.4 m. Skirting was carried out in both winter and summer at approximately 0.6–0.7 m above ground level. Topping was not required during the trial, as the plants remained below 2.5 m in height.

The experiment used young olive trees (cv. Arbosana) planted in SHD system (1429 plants/ha). The irrigation schedule began when the trees were 3 years old. All measurements were made during the first three productive seasons.

### 2.2 Experimental design for irrigation schedule

Irrigation water was supplied to the trees through a self-compensating drip line with a flow rate of 2.3 L/h and emitters spaced 60 cm apart. Irrigation scheduling followed the methodology proposed by Allen et al. (1998), using a simplified water balance approach. Crop evapotranspiration ( ETc) was calculated as:


ETc=ETo×Kc×Kr

where ETo is the reference evapotranspiration obtained from a nearby weather station (Ibba et al., 2023), Kc is the crop coefficient, and Kr is the reduction coefficient for shaded area and was calculated monthly according to Steduto et al. (2012). A Kc value of 0.4 was applied between April and August, as recommended for olive cultivation in La Rioja province (Rousseaux et al., 2008), while a value of 0.68 was used for the remainder of the year (Girona et al., 2002). Until November 2021, plants were irrigated according to full water requirements to promote rapid production onset.

Four deficit irrigation (DI) treatments were evaluated:

- Regulated deficit irrigation 50% (RDI50): During three critical phenological periods - pre-flowering/flowering (PC1), the exponential phase of oil accumulation (PC2), and the period of completion of oil synthesis (PC3) - olive trees received full theoretical water requirements. For the rest of the year, irrigation was reduced to 50% of ETc.
- Regulated deficit irrigation 25% (RDI25): In PC1, trees received full requirements; in PC2 and PC3, 75% of ETc; and during the rest of the year, 25% of ETc.
- Sustained deficit irrigation 70% (CDI70): Trees were irrigated year-round at 70% of theoretical requirements.
- Control: Trees were irrigated year-round at 100% of theoretical requirements (no deficit).

The experimental design consisted of randomized blocks for each irrigation treatment, with four replications. Each plot included 21 trees arranged in a full row, flanked by two adjacent guard rows. Within the central measurement row, a variable number of trees were selected depending on the parameter assessed, excluding the first and last two trees to avoid border effects.

### 2.3 Stem water potential and gas exchange measurements

During the irrigation treatment period, stem water potential ( ${\text{Ψ}}_{stem}$) was measured in five trees per treatment. Measurements were conducted only on clear days, close to solar noon. For each determination, one non-lignified shoot was selected from the inner canopy near the main trunk, bearing six fully expanded, healthy, and shaded leaves. These leaves were pre-covered with moisture-proof aluminum foil bags two hours prior to measurement. Stem water potential was determined following the protocol of Shackel et al. (2000), using a pressure chamber (Bio Control, Argentina) with nitrogen as the inert gas.

Stomatal conductance ( $g_{s}$) was measured on one fully expanded, healthy, sun-exposed leaf per tree, in five trees per treatment. These measurements were taken on the same day and at the same time as the ${\text{Ψ}}_{stem}$determinations, using a leaf porometer (SC1, Decagon Devices, Cambridge, UK) according to the methodology described by Carmassi et al. (2013).

### 2.4 Vegetative growth

Both trunk cross-sectional area growth (measured at 40 cm above ground level) and canopy volume were assessed in 10 trees, following the methodologies described by Caruso et al. (2013). The trunk diameter growth rate was evaluated through the Trunk Cross-Sectional Area (TCSA), a key indicator of vegetative vigor whose restriction is essential in super-high-density (SHD) systems (Pierantozzi et al., 2014; Trentacoste et al., 2015). TCSA was calculated from trunk circumference measurements taken every two weeks at 0.4 m above ground level.

Beginning at bud break, shoot elongation was monitored according to the methodology proposed by Pierantozzi et al. (2014). Measurements were conducted in each cardinal orientation of the plants, using five trees every two weeks, while also recording the number of internodes per shoot.

### 2.5 Fruit measurements and productive parameters

At the beginning of the harvest period (early April), all fruits from the selected trees were manually harvested, and olive production was determined based on fruit yield per tree (kg/tree) and per hectare (kg/ha) for each irrigation treatment. Subsequently, a sample of 100 fruits per replication was taken to determine fruit weight (g) and the maturity index (MI) following the method described by Beltrán et al. (2008). The alternancy index (alternate bearing index), proposed by Hoblyn et al. (1936) and revised by Pearce and Doberšek-Urbanc (1967), was determined to evaluate year-to-year yield fluctuations; this index ranges from 0 to 1, where 0 indicates no alternancy and 1 indicates complete alternate bearing.

Water Productivity (WP) was estimated as the ratio between fresh fruit yield and total water received, as well as oil yield over total water received (Pierantozzi et al., 2013).

#### 2.5.1 Fruit oil content

From each fruit batch, a 100 g aliquot was crushed using a blade mill and dried in an oven until a constant weight was reached. The dried material (1 g) was subjected to continuous and accelerated solid-liquid extraction using a Thermo Scientific EXTREVA ASE Accelerated Solvent Extractor for one hour, with n-hexane as the solvent. Oil content was quantified by the weight difference before and after extraction (AOCS, 1998).

### 2.6 Statistical analysis

Data analysis was performed using InfoStat Professional Software (version 2.0; Di Rienzo et al., 2020). Statistical differences among treatments were evaluated through analysis of variance (ANOVA). Mean comparisons were conducted using Fisher’s LSD test at a significance level of p≤0.05. Relationships among the measured variables were examined using Pearson’s correlation coefficients and regression analysis.

## 3 Results

### 3.1 Environmental conditions of the study site

The study area is characterized by an extremely arid environment, with annual precipitation below 100 mm and high mean temperatures beginning in early spring (**Table 1**). The site exhibits a pronounced thermal amplitude, particularly during spring and winter. Solar radiation levels are among the highest in the country, reaching up to 750 W m^-^² on horizontal surfaces, with mean values in January and February exceeding 600 W m^-^². Under conditions of low atmospheric humidity and high vapor pressure deficit (VPD), local agriculture faces the challenge of extremely high ETo in a context of limited water availability.

**Table 1:** Environmental conditions in Pocito, San Juan – Argentina, across seasons during the period evaluated.

Season		Maximum temperature (°C)	Minimum temperature (°C)	Relative humidity (%)	Rainfall (mm)	Solar radiation (W/m^2^)
2021–2022	Spring	31.4	16.6	51	8.3	545.9
	Summer	32.7	21.5	45	80.9	529.6
	Autumn	22	5.5	55	0	339.1
	Winter	17	1.6	58	0.3	249.2
2022–2023	Spring	30.4	13.7	44	7.9	529.2
	Summer	33.8	19.2	50	32.3	527.2
	Autumn	22.7	9	63	38.2	323.8
	Winter	20.7	3.5	50	0	317.8
2023–2024	Spring	29.5	13.6	43	1.3	500.4
	Summer	35	21.3	49	16.3	535.9
	Autumn	21.1	7.2	61	15.3	329.2
	Winder	16.3	7	49.5	0	310.8

The maximum temperature, minimum temperature, relative humidity, and solar radiation data represent the daily average for each season. Rainfall values represent the accumulation for each season.

The amount of water applied is summarized in **Table 2**. Irrigation volumes increased across all treatments in proportion to vegetative cover, reaching their maximum during the final crop cycle (fifth year after planting). Rainfall was scarce, occurring mainly in December, January, and February, and was virtually absent during the last crop cycle.

**Table 2:** TI, Total irrigation (TI), effective rainfall (ER), total water quantity (TWQ) and reference evapotranspiration (Et0) in the examined olive orchard.

Season	Treatments	TI (mm)	ER (mm)	TWQ (mm)	Et0 (mm)
2021–2022	Control	460.47	32.06	492.53	1314.57
	CDI 70%	322.33	32.06	354.39	1314.57
	RDI 50%	306.01	32.06	338.07	1314.57
	RDI 25%	200.48	32.06	232.54	1314.57
2022–2023	Control	600.73	13.51	614.24	1816.24
	CDI 70%	474.71	13.51	438.22	1816.24
	RDI 50%	418.40	13.51	431.91	1816.24
	RDI 25%	303.02	13.51	316.53	1816.24
	Control	802.63	2.8	805.43	1976.54
2023–2024	CDI 70%	561.84	2.8	564.64	1976.54
	RDI 50%	585.19	2.8	587.99	1976.54
	RDI 25%	425.94	2.8	428.74	1976.54

Considering the total water received (irrigation plus effective precipitation), the control treatment accumulated 492.53 mm during the 2021–2022 season. In comparison, the deficit irrigation treatments achieved water savings of 28.1%, 31.4%, and 52.8% for CDI70, RDI50, and RDI25, respectively. During the second season (2022–2023), the control treatment received 614.24 mm, while savings reached 28.7%, 29.7%, and 48.5% for CDI70, RDI50, and RDI25, respectively. Finally, in 2023–2024, the control treatment received 805.43 mm, with water savings of 29.9%, 27.0%, and 46.8% for CDI70, RDI50, and RDI25, respectively. Overall, the different irrigation strategies maintained relatively stable water-saving percentages across seasons, with CDI70 and RDI50 showing comparable performance.

### 3.2 Plant water relations and gas exchange parameters

**Figure 1:**
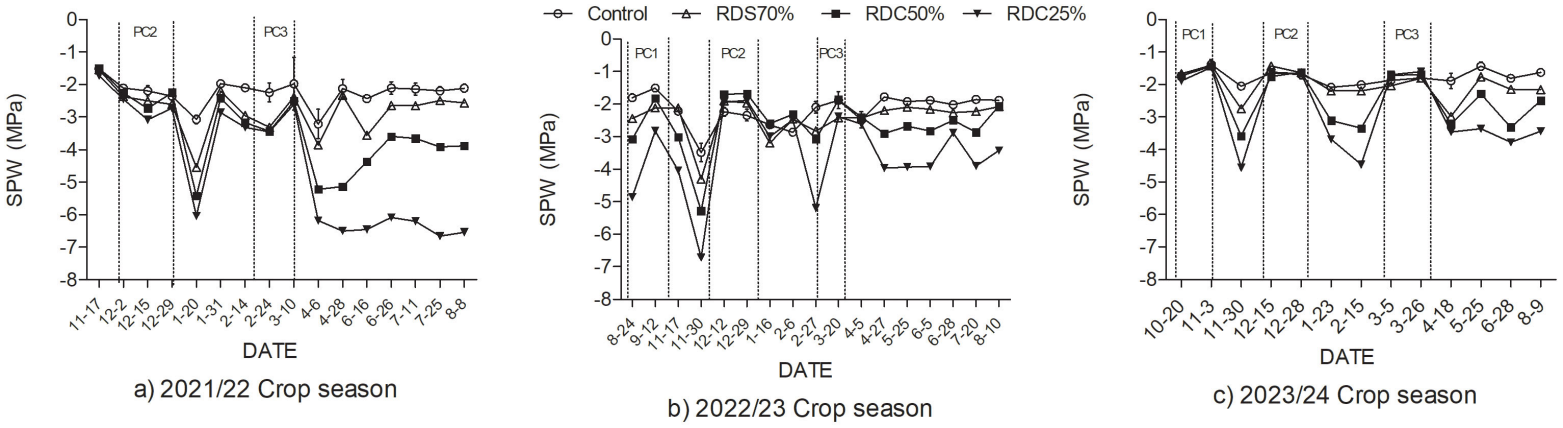
Seasonal variation in water potential of the midday stem (Ψstem) during the 2021/22, 2022/23, and 2023/24 crop cycles **(a–c)**. Irrigation treatments: RDI25% (▼) 25% ETc; RDI50% (◼) 50% ETc; CDI70% (Δ) 70% ETc; Control (O) 100% ETc. Each point represents the average value of 5 independent measurements. The vertical dotted lines indicate the critical periods.

Stem water potential ( ${\text{Ψ}}_{stem}$) is a reliable indicator of olive tree water status (Moriana et al., 2012). **Figure 1** illustrates the evolution of ${\text{Ψ}}_{stem}$ across the three crop cycles under the different irrigation treatments. A similar trend was observed in all cycles tested, with the 2021–2022 and 2022–2023 seasons reaching the lowest values compared to 2023–2024. During the first two seasons, values close to –7 MPa were recorded in the most severe treatment (RDI25). Likewise, during the critical periods PC2 and PC3 in the first two seasons, and PC1 in 2022–2023, both RDI50 and RDI25 exhibited a slow rehydration response, failing to reach values comparable to the control treatment. In contrast, during the 2023–2024 season (**Figure 1c**), ${\text{Ψ}}_{stem}$ values in these DI treatments approached those of the control, indicating rapid rehydration, despite RDI25 receiving lower water inputs in PC2 and PC3 relative to the control and RDI50. The CDI70 treatment showed stable performance throughout the three crop cycles, consistently remaining slightly below the Control.

**Figure 2:**
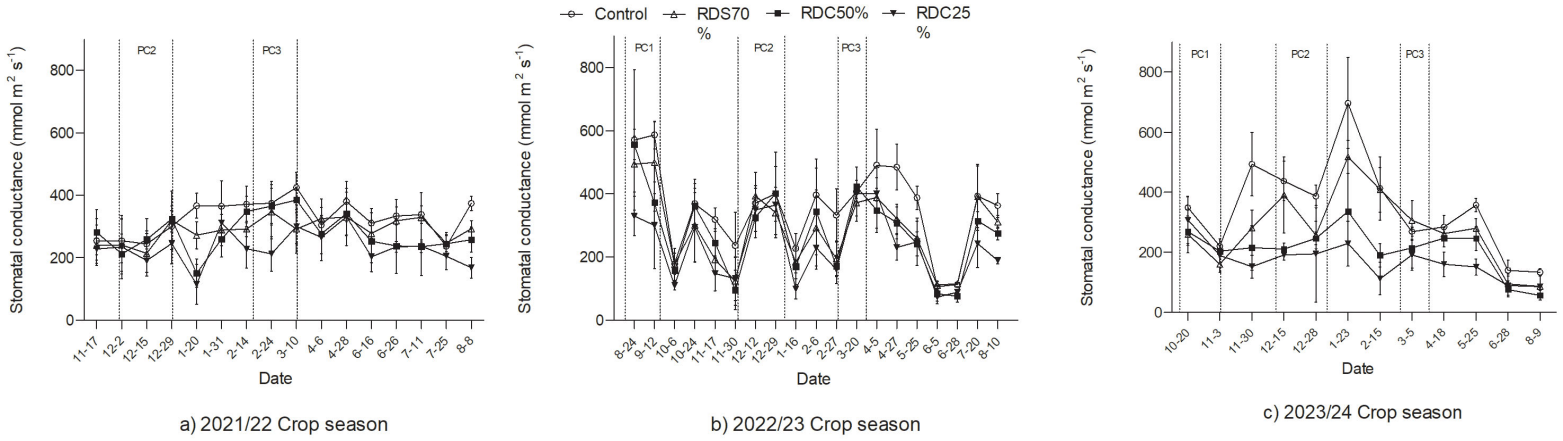
Stomatal conductance (gs) throughout the 2021/22, 2022/23, and 2023/24 growing seasons **(a–c)**. Irrigation treatments: RDI25% (▼) 25% ETc; RDI50% (◼) 50% ETc; CDI70% (Δ) 70% ETc; Control (O) 100% ETc. Each point represents the average value of 5 independent measurements. The vertical dotted lines indicate the critical periods.

Stomatal conductance ( $g_{s}$) is another key physiological parameter, as its reduction represents the primary mechanism by which olive trees limit transpiration under water deficit conditions (Moriana and Fereres, 2003). The first differences among treatments were detected at measurement 5 (after PC2), when RDI50 and RDI25 showed the lowest values compared to the Control (**Figure 2a**). Overall, $g_{s}$ followed the same pattern as ${\text{Ψ}}_{stem}$, with more pronounced differences between deficit treatments and the Control (**Figure 2**). Across both seasons, RDI50 and RDI25 consistently exhibited lower values than the Control and CDI70. Furthermore, in 2022–2023, all treatments recorded lower values than in 2021–2022, while the 2023–2024 season showed generally higher $g_{s}$ values, consistent with the ${\text{Ψ}}_{stem}$ results.

### 3.3 Vegetative growth

**Figure 3:**
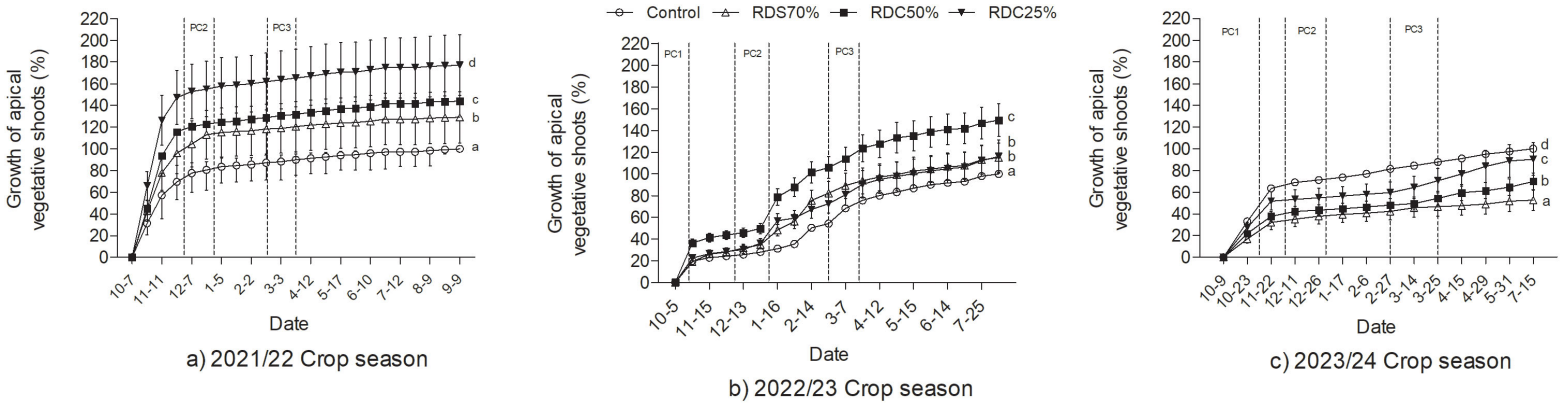
Growth of apical vegetative shoots (%) measured across three growing seasons (**a**, 2021/22; **b**, 2022/23; **c**, 2023/24). Irrigation treatments: RDI25% (▼) 25% ETc; RDI50% (◼) 50% ETc; CDI70% (Δ) 70% ETc; Control (O) 100% ETc. Each point represents the average value of 40 independent measurements. The vertical dottedlines indicate the critical periods.

One of the primary objectives of deficit irrigation in olive groves intended for harvesting with over-the-row machinery is to limit vegetative vigor (Pierantozzi et al., 2020). In the first productive cycle, all treatments showed a rapid increase in shoot length during the initial 60 days of measurement, after which growth continued at a slower rate throughout the remainder of the season (**Figure 3a**). In the second year, growth followed a typical sigmoidal pattern (**Figure 3b**). Notably, the RDI50 treatment exhibited 49% greater shoot elongation compared to the Control. In the final cycle, when hedgerows were approaching closure, the impact of deficit irrigation became more evident (**Figure 3c**). Treatments under DI showed significant reductions in shoot growth relative to the Control. Specifically, CDI70 resulted in 52% less growth, while RDI50 and RDI25 showed reductions of 31% and 10%, respectively.

Variations in trunk diameter growth and internode number and length are shown in **Table 3**. During the 2021–2022 season, the control and RDC25treatments exhibited the greatest growth increments. In 2022–2023, the RDC50 treatment produced 50% higher growth than the other treatments, including the control. In the following season (2023–2024), the CDI70 and RDC50 treatments showed significantly lower values compared to the control, with no differences between them, whereas the RDC25 strategy resulted in values similar to those of the control.

**Table 3:** Trunk diameter growth (TD), average number and length of internodes (ANI and LI, respectively) of olive trees grown under different water irrigation treatments.

Parameter	Treatment	2021-2022	2022-2023	2023-2024
TD (mm)	Control	6.32 ± 0.46ab	4.99 ± 0.24 a	4.2 ± 0.57 b
	CDI 70%	4.88 ± 0.44a	5.52 ± 0.34 a	1.56 ± 0.35 a
	RDI 50%	4.97 ± 0.56a	7.5 ± 0.54 b	0.98 ± 0.15 a
	RDI 25%	7.83 ± 1.24b	5.51 ± 0.73 a	3.17 ± 0.45 b
ANI	Control	3.10 ± 0.3a	4.49 ± 0.54a	6.07 ± 0.91b
	CDI 70%	2.8 ± 0.28a	4.58 ± 0.39a	2.08 ± 0.29a
	RDI 50%	3.09 ± 0.21a	5.68 ± 0.4b	2.96± 0.32a
	RDI 25%	3.09 ± 0.21a	4.54 ± 0.27ab	4.79 ± 0.41b
LI (mm)	Control	20.08 ± 1.75a	8.04 ± 0.53ab	15.68 ± 2.49b
	CDI70%	20.24 ± 2.02a	7.68 ± 0.43ab	10.29 ± 1.16ab
	RDI 50%	24.92 ± 1.39ab	8.94 ± 0.40b	8.53 ± 3.0a
	RDI 25%	29.56 ± 1.88b	7.12 ± 0.42a	11.72 ± 0.49ab

For each crop season, data are the average (± standard error) of 40 independent measurements.

Data are the average of three crop seasons. Irrigation treatments: CDI70% (70% of ETc), RDI50% (50% of ETc), RDI25% (25% of ETc), control (full-irrigated, 100% of ETc).

Mean values with different superscript letters present significant differences (P £ 0.05) among irrigation treatments in each crop season.

The number of internodes exhibited a pattern similar to that of shoot growth, although with some differences during the first year during which no significant differences were observed among the irrigation treatments. In the second season, the RDC50 strategy resulted in a 27% increase relative to the control. Finally, in the last season, both the CDI70 and the RDC50 treatments significantly reduced the number of internodes, consistent with the observed shortening in shoot length. Internode length followed a pattern comparable to the other parameters, with pronounced annual variations between seasons.

**Figure 4:**
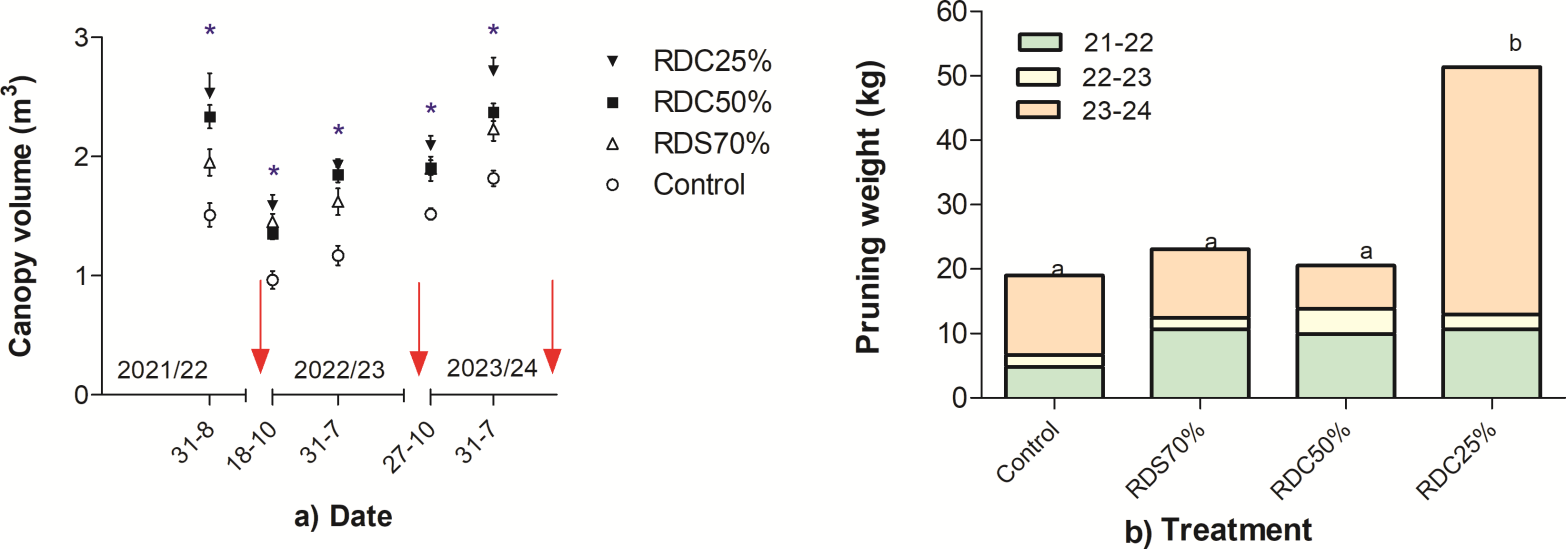
**(a)** Canopy volume (m^3^) across three growing seasons. The red arrows indicate when mechanical pruning was performed. * indicate significant differences (P ≤ 0.05) among irrigation treatments. **(b)** Pruning weight (kg) during 2021/22 (green), 2022/23 (yellow) and 2023/24 (orange) growing periods. Different superscript letters present significant differences (P ≤ 0.05) among irrigation treatments in accumulate crop season. Irrigation treatments: RDI25% (▼) 25% ETc; RDI50% (◼) 50% ETc; CDI70% (Δ) 70% ETc; Control (O) 100% ETc. Each point represents the average value of 10 independent measurements.

**Figure 4a** reports the canopy volume across the three studied crop seasons. Despite the pruning performed, a significant effect was observed starting from the first year in the most stressed treatments (RDC50 and RDC25); surprisingly, these exhibited the highest canopy volume values in each season, relative to the control. As a consequence of this increase, the treatments with the highest water deficits were those from which the largest amount of plant material was extracted as pruning residue over the total of the three seasons (**Figure 4b**).

### 3.4 Productive parameters

Yield, expressed either as fruit or oil production, was influenced by both the amount of water applied and the irrigation strategy used (**Table 4**). Cumulative fruit yield over the first three growing years was lower in the more stressful treatments: RDI50 and RDI25 yielded 13% and 72% less fruits, respectively, than the control. Differently, the CDI70 treatment showed a 13% increase relative to the control.

**Table 4:** Yield parameters of olive trees grown under different water irrigation treatments.

Crop season	Treatment	Fruit weight (g)	Fruit load (fruit number/tree)	Fruit yield (kg/ha)	Oil yield (kg/ha)	WP (kg fruit/m^3^)	Maturity index	Alternancy index
2021–2022	Control	1.89 ± 0.06^cB^	1970 ± 192.04^aA^	4844 ± 427.07^aA^	923.5 ± 88.8^aA^	1.03 ± 0.09^aA^	2.78 ± 0.05^aC^	
	CDI 70%	1.67 ± 0.08^bA^	3158 ± 255.81^bA^	7058 ± 342.42^cA^	1407 ± 77^bA^	1.99 ± 0.10^bB^	3.17 ± 0.11^bB^	
	RDI 50%	1.06 ± 0.03^aA^	3880 ± 172.91^cB^	5866 ± 302.32^bA^	1313 ± 66.59^bB^	1.73 ± 0.09^bB^	4.23 ± 0.10^cB^	
	RDI 25%	0.97 ± 0.01^aB^	3948 ± 273.62^cB^	5440 ± 354.71^abB^	1220 ± 74.5^bB^	2.44 ± 0.16^cB^	4.48± 0.12^cC^	
2022–2023	Control	1.54 ± 0.02^bA^	3390 ± 147.15^cC^	7355 ± 348.79^cB^	1441 ± 77.3^bB^	1.20 ± 0.06^bB^	2.38 ± 0.03^aB^	0.20
	CDI 70%	1.94 ± 0.10^cB^	2559 ± 317.81^bA^	5231 ± 723.36^bB^	1173 ± 165^bA^	1.19 ± 0.17^bA^	3.05 ± 0.19^cB^	0.14
	RDI 50%	1.52 ± 0.13^bB^	714 ± 345.16^aA^	874 ± 394.68^cB^	187.5 ± 81.74^aA^	0.20 ± 0.09^aA^	4.23 ± 0.11^bB^	0.74
	RDI 25%							1
2023–2024	Control	2.18 ± 0.04^bC^	2609 ± 214.22^bB^	7916 ± 637.51^bB^	1670 ± 143.17^bB^	0.98 ± 0.08^bA^	2.06 ± 0.07^aA^	0.03
	CDI 70%	1.55 ± 0.08^aA^	5088 ± 171.42^cB^	10390 ± 510.18^cC^	2074 ± 100^cB^	1.84 ± 0.09^cB^	2.27± 0.02^bA^	0.33
	RDI 50%	0.98 ± 0.03^aA^	7717± 141.46^dC^	10673 ± 420.99^cC^	2170 ± 97^cC^	1.82 ± 0.07^cB^	2.47 ± 0.05^cA^	0.84
	RDI 25%	1.23 ± 0.41^aB^	349 ± 395.38^aA^	227 ± 193.13^aA^	39.51 ± 33.34^aA^	0.35 ± 0.05^aA^	3.01 ± 0.14^dB^	0.91
**Accumulated**	**Treatment**			**Fruit yield** **(kg/ha)**	**Oil yield** **(kg/ha)**	**WP** **(kg fruit/m^3^)**	**WP** **(kg oil/m^3^)**	
	Control			20115 ± 1194^c^	4034 ± 269^b^	1.05 ± 0.06^b^	0.21 ± 0.01^b^	
	CDI 70%			22679 ± 841^d^	4654 ± 176^c^	1.67 ± 0.06^d^	0.34 ± 0.01^d^	
	RDI 50%			17413 ± 785^b^	3671 ± 168^b^	1.28 ± 0.06^c^	0.27 ± 0.01^c^	
	RDI 25%			5668 ± 426^a^	1259 ± 86^a^	0.59 ± 0.04^a^	0.13 ± 0.01^a^	

For each crop season, data are the average (± standard error) of 40 independent measurements. Mean values with different superscript small letters present significant differences (P £ 0.05) among irrigation treatments in each crop season. Mean values with different superscript capital letters present significant differences (P £ 0.05) among crop seasons in each irrigation treatment.

Data are the average of three crop seasons. Irrigation treatments: CDI 70% (70% of ETc), RDI 50% (50% of ETc), RDI 25% (25% of ETc), Control (full-irrigated, 100% of ETc). WP, water productivity.

Crop load also exhibited significant variations across seasons. In the first year, all DI treatments increased crop load: relative to the control treatment, increments were about 68% (CDI70) and more than 100% (both RDC treatments). In the following year (2022–2023), the opposite pattern was observed: the DI treatments showed significant reductions in crop load relative to the control treatment. Of note, however, were the results from the 2023–2024 crop season where no significant differences were found between both the RDI50 and the CDI70 treatments, which produced 30% more fruits relative to the control treatment.

On the other hand, important variations were observed in regarding cumulative fruit and oil yields over the three years. The CDI70 treatment produced the highest fruit and oil yields, followed by the control, RDI50 and RDI25 treatments. In this latter yielded were about 70% lesser than that of the control. As expected, fruits from the control treatment were generally larger than those from the DI treatments. Only in the second crop cycle the CDI70 produced heavier fruits than the control, with RDI50 showing a similar trend.

Water productivity (WP) varied depending on the crop year considered. In the first crop cycle, it was higher in the most restrictive irrigation treatment (RDI25). In the second cycle, both the control and CDI70 treatments showed remarkable increments in WP, while both RD treatments presented negligible values. In the third cycle, both the CDI70 and the RDI50 treatments reached similar WP values, which almost doubled the value observed in the control treatment. Meanwhile, the RDI25 treatment did not show satisfactory recovery, remaining at values well below those of the control group. Considering the three productive seasons as a whole, the ranking of WP was CDI70 > RDI50 > Control > RDI25. The same order was observed for oil−based WP (data not shown).

The maturity index (MI) increased significantly in all DI treatments relative to the control. Considering the three crop years evaluated, it reached average values close to 3 (CDI70) or greater than 3.5, while it was 2.4 in the control treatment.

## 4 Discussion

During the crop cycles evaluated, precipitation was concentrated mainly in the summer and was clearly insufficient to offset the crop’s evapotranspirative demand of the crop. In this sense, this study is novel in that it analyzes multiple levels of water stress under conditions where the crop’s water supply depends almost entirely on irrigation throughout the entire growing period.

The values of ${\text{Ψ}}_{stem}$ declined progressively as irrigation treatments became more restrictive, with clear differentiation among all treatments. However, the control treatment exhibited values near or below 2 which are lower than those expected for a non-water stressed crop, particularly during the first two cycles. Values near −3 MPa are typically considered indicative of severe water stress, as reported by Moriana et al. (2012). Although stem water potential values from Central West and Northwest Argentina may differ slightly from those measured in other environments, the occurrence of such low values in the control treatment suggests that the Kc values applied in our trial may have been insufficient to sustain the water status of a fully irrigated plant (Pierantozzi et al., 2020). A complementary hypothesis is that, because these low values occurred during the first two cycles, the Kr factor may not have accurately estimated ETc under conditions of limited ground cover. This is relevant to the present study which covers years three to five of the establishment period of an orchard. Here, Kr was calculated following the methodology proposed by Steduto et al. (2012). However, other authors prefer the approach of Fereres and Castel (1981), also recommended by Orgaz et al. (1999). In all these approaches, Kr remains an empirical coefficient that relates the ETc of an incompletely covered orchard to that of a mature canopy.

It is also important to note that these coefficients were originally developed for crops other than olives and rely solely on the horizontal projection of the canopy, without accounting for differences in leaf area density that may create gaps within the canopy shadow. Furthermore, these relationships were established for trees of diverse shapes, grown as isolated individuals with spherical or conical canopies conditions that differ substantially from modern olive production systems, including SHD orchards.

The *g_s_* is a key physiological variable for optimizing plant water use under drought conditions (Giorio et al., 1999). Stomata operate through highly regulated mechanisms that balance the plant’s requirement for CO_2_ assimilation with the need to limit transpiration water loss according to prevailing environmental constraints (Díaz-Espejo, 2000). The different irrigation strategies could be clearly differentiated by following the trends in Ψ-stem values which in turn had a significant association with *g_s_* (Pearson’s coefficient r = 0.32, p < 0.01). Under Continuous Deficit Irrigation (CDI), where water restriction was imposed uniformly throughout the growing season, the decline in *g_s_* was moderate but sustained, indicating a stable physiological adjustment to prolonged water restriction. In contrast, Regulated Deficit Irrigation (RDI) induced a sharper reduction in *g_s_* during the designated stress phase (RDI25 and RDI50), followed by a rapid recovery upon re-watering. These responses align with previous reports describing the dynamic stomatal behavior under controlled water deficits in olive orchards (Agüero Alcaras et al., 2016; Iglesias, 2023). Moderate declines in *g_s_* associated with mild water stress often do not constrain photosynthetic rates, although they may reduce vegetative growth (Bradford and Hsiao, 1982), which could be due to relocation of carbohydrate resources toward reproductive development. Conversely, severe irrigation restrictions, such as those in the RDI25 treatment, frequently trigger substantial stomatal closure and pronounced declines in leaf water potential, particularly during periods of peak evaporative demand (Alegre et al., 1999; Moriana and Fereres, 2003). Collectively, these stomatal responses represent an effective physiological strategy for enhancing Water Use Efficiency (WUE). By moderating transpiration water loss while maintaining sufficient carbon assimilation, plants can sustain productivity under deficit irrigation without causing major reductions in total oil yield (Agüero Alcaras et al., 2016).

For the first crop cycle, the monomolecular model provided the best fit for shoot growth, consistent with models reported by other authors (Costanza et al., 2025). Statistically significant differences were recorded between treatments for maximum growth achieved, while no differences were found in growth rates. The response of the trees in the first year contrasts with results from other studies, where an increase in water stress corresponds to a decrease in growth (Pierantozzi et al., 2020). For the second crop cycle, the double logistic model provided the best fit, suggesting a shift in growth dynamics toward a sigmoidal pattern more typical of rainfed olives, where resources limit growth in the final stages (Pierantozzi et al., 2014). This would reinforce the idea that during this second year, all treatments may have experienced some degree of water stress, likely due to an underestimation of ETc (crop evapotranspiration) possibly caused by an imprecise estimation of Kr or Kc. Finally, in the third crop cycle, shoot growth again adjusted to a monomolecular model. Statistically significant differences were once more recorded between treatments for maximum growth, with no differences in growth rates. The treatments showing the major growth were the most extreme ones (Control and RDI25), making it evident that other factors play a more preponderant role in shoot growth than water supply alone.

**Figure 5:**
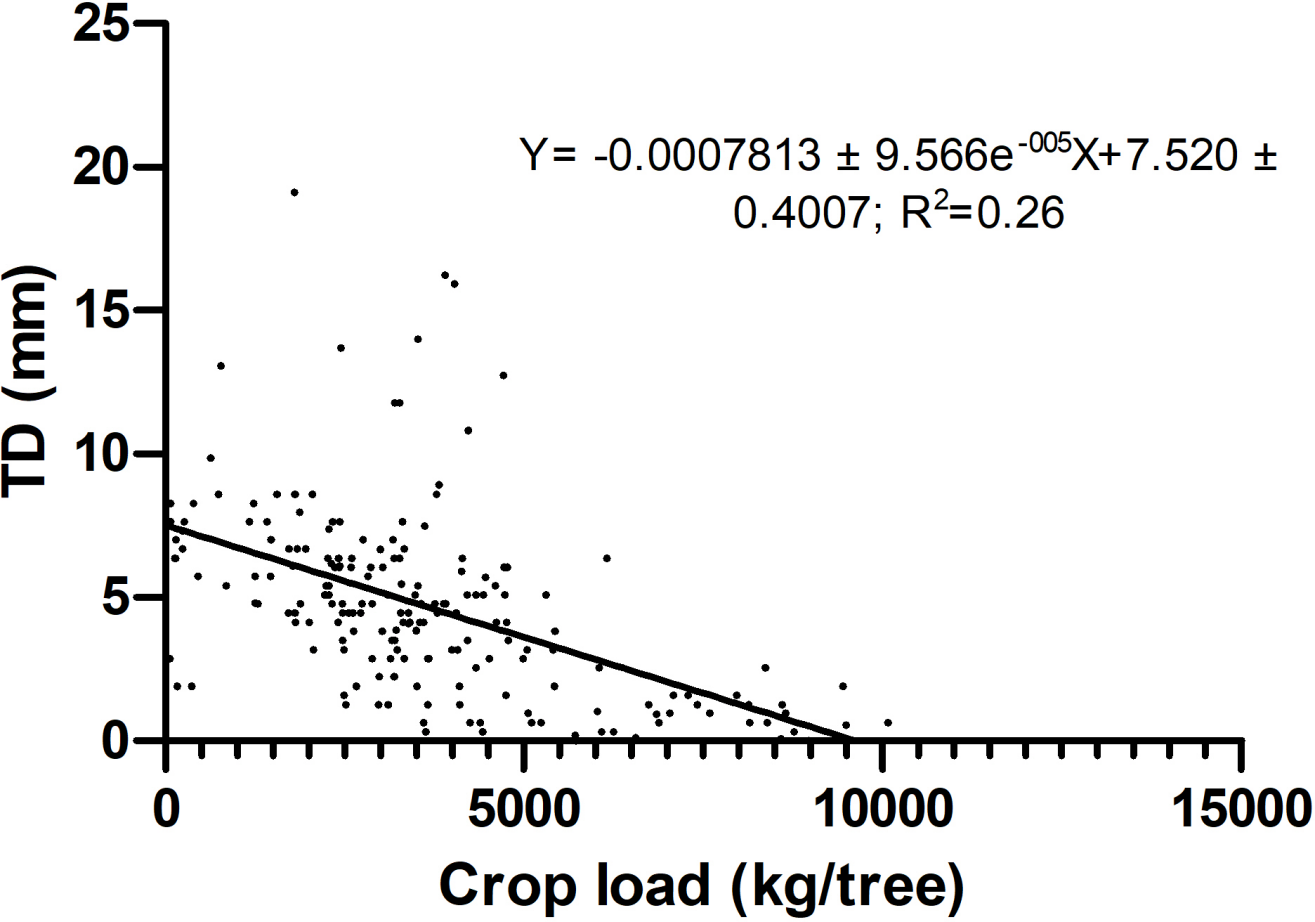
Variation of trunk diameter growth (mm) and crop load (kg/tree). Data correspond to all crop seasons examined. Each point represents one olive tree. Y= -0.0007813 ± 9.566e-005X+7.520 ± 0.4007; R^2^ = 0.2.6.

Regarding trunk diameter growth, the responsiveness of trunk diameter variations to deficit irrigation has long been recognized in olive trees (Agüero Alcaras et al., 2016). In our study, trunk diameter significantly increased in well-watered control trees and in those of the most stressed treatment (RDI25) during the first and last crop cycles. Only in the 2022–2023 cycle greater growth was found in the RDI50 treatment, specifically when this treatment had no fruit load. In this regard, similar increases throughout the growing season have been observed in other studies for trees with low crop loads (Moriana and Fereres, 2003; López-Bernal et al., 2015). Tree productivity is a key determinant of vegetative growth and must therefore be considered when interpreting trunk expansion dynamics. A regression analysis between trunk diameter growth and fruit load revealed a strong negative linear relationship. This outcome should be interpreted in the context of the considerable variability encompassed in the dataset, including differences in plant water status, contrasting irrigation strategies (CDI and RDI), the multiple seasons analyzed, and the young age of the trees, evidenced by the progressive increase in canopy volume across years (**Figure 5**).

When correlating integrated stem water potential with trunk growth, the relationship remained statistically significant (P < 0.05), though the corresponding R^2^ was markedly lower (data not shown). **Table 5** presents the Pearson correlation coefficients among vegetative growth variables, fruit load, and integrated stem water potential across the different seasons. These results clearly indicate that fruit load exerts a dominant influence on the vegetative parameters examined.

**Table 5:** Pearson correlation coefficients (r) among fruit load and trunk diameter growth (TD), canopy volume (CV), apical vegetative growth (AVG) and integrated stem water potential (ISWP) across the different seasons.

Season	Parameters	r
2021–2022	TD	-0.29
	CV	0.57^*^
	AVG	0.18
	ISWP	0.55^*^
2022–2023	TD	-0.44^*^
	CV	-0.55^*^
	AVG	-0.50^*^
	ISWP	-0.71^*^
2023–2024	TD	-0.66^*^
	CV	0.28^*^
	AVG	-0.52^*^
	ISWP	0.44^*^

*indicate significant differences (P ≤ 0.01).

In a previous study, Pierantozzi et al. (2020) demonstrated that spring water restriction strongly affects vegetative growth. However, when water deficits occur throughout the entire crop cycle, other factors related to productivity and the allocation of photoassimilates become increasingly relevant. The critical role of fruit load in olive tree physiology is well established (Lavee, 2015). In fact, several authors have evaluated the application of differential irrigation strategies during “on” (high yield) and “off” (low yield) years in olive orchards, though results have been inconsistent (Moriana and Fereres, 2003; Ben-Gal et al., 2021). Our results suggest that to control vigor in young, actively growing trees in an SHD olive orchard, production is essential; any irrigation strategy implemented must maintain high production to be effective.

The number of internodes was linearly correlated with shoot length across all three cycles and the four treatments considered (y=0.05 ± 0.0024 + 2.06 ± 0.15, r^2^ = 0.63). This strong functional dependence is a characteristic trait of olive morphology, indicating that any factor (such as deficit irrigation) affecting the longitudinal shoot growth rate will simultaneously affect the rate of node formation. We found that irrigation was not effective in controlling internode length, as variations between cycles were much larger than any variation between treatments. Rosati et al. (2013) have suggested that internode length and shoot length are not specific characteristics of cultivars better adapted to SHD. Instead, greater branching per unit of trunk (or shoot) length, along with a lower growth rate in trunk and branch diameter, have been consistently identified as effective indicators for cultivars and rootstocks best suited for high density (Rosati et al., 2024). These traits contribute to reduced or controllable vigor, allowing tree height and canopy volume to remain within the limits imposed by the hedgerow, thereby facilitating mechanical harvesting (Gómez del Campo et al., 2015).

Fruit yield, oil fruit content, and the expression of alternate bearing are central outcomes in any irrigation study. In the first crop cycle, the results were unexpected: the RDI50 and CDI70 treatments produced the highest fruit yields (kg/ha). This was primarily driven by a greater fruit load in the water restricted treatments, particularly under the RDI regimes. In olive, as in other drupaceous species, the number of fruits per tree is the main determinant of yield, while individual fruit weight plays a secondary role (Lavee and Wodner, 1991).

Oil yield (kg/ha) followed the same pattern than fruit yields, with differences among treatments explained largely by fruit yield rather than by a direct effect of water supply. Oil content on a fresh weight basis was higher under the most water restricted treatments, a trend associated with the stage of ripeness at harvest. With some differences between the different olive varieties, most studies indicate that oil accumulation is essentially complete when the Maturity Index (MI) is between 3 – 4, corresponding to fruits in transition to veraison, with some of them displaying black skin (Torres and Maestri, 2006; Beltrán et al., 2008). This is consistent with the patterns observed in the present study.

Fruit ripening is known to accelerate under lighter crop loads and is also strongly promoted by moderate water stress (Beltrán et al., 2008). Accordingly, in our study the highest veraison levels at harvest occurred in the most water restricted treatments in all three crop cycles, even though variations in crop load further intensified these effects. Conversely, individual fruit weight was greater under higher water supply, a response also amplified by lower crop loads. These relationships are widely documented in olive (Fernández and Moreno, 2000; Gucci et al., 2012). In the second crop cycle, production in the RDI treatments was nearly zero. This is consistent with the well-known alternate bearing behavior of olive trees, where a low yield year typically follows a high yield year (Lavee and Avidan, 1994). In contrast, yield under the CDI treatment remained comparable to the Control. By the third cycle, RDI50 and CDI70 again produced higher yields than the Control, whereas the most stressed treatment exhibited another year of near zero production. While the low yield in the second year is readily attributed to alternate bearing, a second consecutive year of minimal production suggests a possible stress memory effect, in which plants “remember” previous stress events and mount a stronger response to subsequent ones (Fleta-Soriano and Munné-Bosch, 2016). Regardless of the terminology alternate bearing, stress legacy, or stress memory, the results clearly show that water deficit in one season influences performance in the next.

Across the three cycles, the CDI70 treatment (moderate, sustained deficit) produced a higher cumulative yield than RDI50. Although CDI70 does not reach high production peaks – possibly due to continuous restrictions on vegetative and reproductive growth - it could promote a stable balance which, as suggested by Corell et al. (2020), could reflect physiological acclimation to persistent water limitation.

Total yield varied substantially between “on” and “off” years. In line with findings by Gucci et al. (2019), RDI50 may have provided recovery windows that enhance cell expansion when water potential is restored, thereby maximizing production in favorable years but inducing sharp declines in the following season. In high productivity years, RDI50 achieved the highest fruit counts (**Table 4**). The strong alternate bearing observed under RDI50 is consistent with the physiological basis of the phenomenon: fruit presence - particularly seeds – may have inhibited floral induction through hormonal signaling processes (Lavee and Avidan, 1994; Fabbri and Gucci, 2023). The combination of high fruit numbers and reduced shoot growth under RDI50 ultimately amplified production peaks and increased the alternate bearing index (**Table 4**). In contrast, the “load regulation” imposed by a moderate, steady deficit such as CDI70 could support more uniform yields across years. Rather than producing extreme peaks, CDI70 could foster sustainable, long term production with reduced oscillation between “on” and “off” years.

## 5 Conclusions

This study provides novel insights regarding responses to irrigation strategies of young olive trees in a SHD system under arid, non-Mediterranean climate. Main findings indicate that the choice of a deficit irrigation strategy is critical for ensuring productive stability and long term sustainability. Among the treatments evaluated, Sustained Deficit Irrigation at 70% ETc (CDI70) emerged as the most effective approach. It provided an optimal balance between substantial water savings (approximately 30%) and the highest cumulative fruit and oil yields. In contrast to Regulated Deficit Irrigation strategies (RDI50 and RDI25) - which induced severe water stress and amplified the cultivar’s natural alternate bearing tendency - CDI70 promoted stable physiological acclimation that supported consistent production across three consecutive seasons.

The study also shows that in young, SHD hedgerow systems, vegetative vigor is driven more by reproductive load than by irrigation level alone. The strong negative relationship observed between trunk diameter growth and fruit load indicates that maintaining high yields is essential for controlling canopy development in these intensive systems. Although the RDI treatments (RDI50 and RDI25) achieved greater short term water savings, they resulted in drastic yield reductions in subsequent years, suggesting a “stress memory” or legacy effect that compromises orchard resilience. Additionally, all deficit irrigation treatments accelerated fruit ripening, highlighting a potential management tool for optimizing harvest timing.

In summary, for the first three productive years of a young hedgerow orchard, CDI70 may be a sustainable irrigation strategy. This irrigation strategy was found to give appropriate balance between water conservation and the productive regularity required for mechanized SHD systems. Nevertheless, it will be necessary to evaluate this strategy over the longer term.

Another aspect to consider in future research relates to the use of traditional crop coefficients (Kc and Kr) in young olive orchards located in extremely arid environments. For physiological and stem water potential measurements, such coefficients may require better calibration to avoid unintended stress in well-irrigated control treatments.

## Data Availability

The original contributions presented in the study are included in the article/supplementary material. Further inquiries can be directed to the corresponding author.
